# Mitochondrial DNA Instability and Metabolic Shift in Human Cancers

**DOI:** 10.3390/ijms10020674

**Published:** 2009-02-23

**Authors:** Hsin-Chen Lee, Yau-Huei Wei

**Affiliations:** 1 Institute of Pharmacology, National Yang-Ming University, Taipei, Taiwan 112; E-Mail: hclee2@ym.edu.tw; 2 Department of Biochemistry and Molecular Biology, National Yang-Ming University, Taipei, Taiwan 112

**Keywords:** Cancer, Mitochondrial DNA, Somatic mutation, Metabolic shift, Genome instability

## Abstract

A shift in glucose metabolism from oxidative phosphorylation to glycolysis is one of the biochemical hallmarks of tumor cells. Mitochondrial defects have been proposed to play an important role in the initiation and/or progression of various types of cancer. In the past decade, a wide spectrum of mutations and depletion of mtDNA have been identified in human cancers. Moreover, it has been demonstrated that activation of oncogenes or mutation of tumor suppressor genes, such as p53, can lead to the upregulation of glycolytic enzymes or inhibition of the biogenesis or assembly of respiratory enzyme complexes such as cytochrome *c* oxidase. These findings may explain, at least in part, the well documented phenomena of elevated glucose uptake and mitochondrial defects in cancers. In this article, we review the somatic mtDNA alterations with clinicopathological correlations in human cancers, and their potential roles in tumorigenesis, cancer progression, and metastasis. The signaling pathways involved in the shift from aerobic metabolism to glycolysis in human cancers are also discussed.

## 1. Introduction

Mitochondria are cytoplasmic organelles that play a variety of important roles, including the generation of ATP through respiration and oxidative phosphorylation (OXPHOS), production of reactive oxygen species (ROS), and initiation and execution of apoptosis [1]. Compared with glycolysis, mitochondrial respiration and OXPHOS is a more efficient pathway to generate ATP from glucose in normal human cells. In contrast, a shift in glucose metabolism from OXPHOS to glycolysis has been frequently observed in cancer cells and represents an important biochemical hallmark of tumors.

As early as 1930, the German biochemist Otto Warburg first proposed that tumor cells, unlike normal cells, exhibit an increased utilization of glycolytic pathway for energy production, even when there is abundant oxygen for mitochondria to make ATP via respiration and OXPHOS [2]. This phenomenon is known as the Warburg effect. He further proposed that defects in energy metabolism, specifically in mitochondria, may play an important role in the initiation or progression of cancers [3]. Based on the fact that glucose uptake is increased in cancer cells, the positron emission tomography (PET) of ^19^F-glucose in affected tissues has been widely used in clinical diagnosis of cancers. However, the molecular and cellular mechanisms of the Warburg effect and its roles in cancer biology have not been fully elucidated.

Human mitochondrial DNA (mtDNA) is a 16,569 bp double-stranded circular DNA molecule, and several hundred to several thousand copies of mtDNA are present in each cell. The human mtDNA encodes 13 polypeptides, which are essential constituents of respiratory enzyme complexes, and 22 transfer RNAs and two ribosomal RNAs that are required for protein synthesis in mitochondria [4]. Mammalian mtDNA is more susceptible to oxidative damage and has a higher mutation rate compared with nuclear DNA due to a lack of protective histone proteins, limited DNA repair activities, and a high rate of generation of ROS in mitochondria [5]. Somatic mutation and damage to mtDNA can result in impairment of the OXPHOS system and enhanced ROS production, which in turn accelerates the rate of DNA mutation. This scenario has been proposed to be involved in the initiation of carcinogenesis [6].

One of the hypotheses, which may explain the Warburg effect, is that cancer cells accumulate mtDNA alterations and lead to defects in mitochondrial respiration and ATP generation by the OXPHOS system [7,8]. It has been established that some of the acquired mtDNA mutations can impair the function of OXPHOS, increase the production of ROS and thereby promote tumor cell proliferation. The other acquired mutations of mtDNA may confer tumor cells with the ability to adapt to the new microenvironment or cope with stress during progression and metastasis of the tumor.

In this article, we review recent findings of somatic mtDNA alterations and mitochondrial dysfunction in human cancers. Moreover, we examine the roles of mutation and depletion of mtDNA in the pathophysiology, initiation and progression of cancers. The signaling pathways that may be involved in the shift of aerobic metabolism to glycolysis in cancer cells are also discussed.

## 2. Somatic mtDNA alterations in human cancers

In the past decade, various types of mtDNA alterations have been identified in primary human cancers. These mtDNA alterations include point mutations, deletions, insertions, tandem duplications, and copy number changes.

### 2.1. Point mutations

Polyak *et al.* [9] first reported that somatic point mutations of mitochondrial genome occurred in human colorectal tumors. In this study, the entire mtDNAs of 10 human colorectal cancer cell lines were completely sequenced and seven were found to carry mutations in protein coding genes or rRNA genes. Importantly, the study further revealed that most of the mtDNA mutations were homoplasmic. On the basis of these findings, the authors suggested that mitochondria could rapidly become homogeneous in colorectal cancer cells. Moreover, some of these point mutations identified within protein-coding regions could lead to frame-shift or amino acid substitutions.

Fliss *et al.* [10] reported that 64% (9/14) of bladder cancers, 46% (6/13) of head and neck cancers, and 43% (6/14) of lung cancers harbored point mutations of mtDNA. It was confirmed that the majority of these somatic mutations of mtDNA were homoplasmic. In addition to mutations in the coding region of mtDNA, a high frequency of somatic mutation was located in the non-coding displacement loop (D-loop) region of mtDNA.

Table 1 summarizes the results of recent studies on primary tumors [9–25]. The data clearly indicate that high frequencies of somatic mutations of mtDNA occur in various types of cancers, and that many of the mtDNA mutations are located in the D-loop region of mtDNA. A number of extensive analysis of somatic mutation in the D-loop region of mtDNA revealed that base insertions or deletions at nucleotide position (np) 303–309, a polycytidine stretch (C-tract) termed D310, are the most common mutations of mtDNA in human cancers (Table 2) [10–16,18,19,21–23,25–46]. Some of the D310 variations have also been reported as common variants in normal human tissues [47].

An *in vitro* analysis revealed that the D-loop, and especially the D310 region, is more susceptible to oxidative damage and electrophilic attack as compared with other regions of mtDNA [48]. In addition to a high susceptibility to DNA damage and mutation, an inefficient DNA repair system in mitochondria has been suggested to contribute to the high frequency of homoplasmic D310 C-tract frame-shift mutations in many types of cancers [48]. Extensive oxidative damage to the poly C repeat may result in slipping and/or mis-incorporation during replication or repair of mtDNA by mitochondrial DNA polymerase γ, and in turn lead to mtDNA mutations in cancer cells. In many human cancers the decrease in the replication and DNA repair activities of DNA polymerase γ could contribute to the extremely high incidence of mutation in the D-loop of mtDNA [49,50].

### 2.2. Deletions

Large-scale deletions of mtDNA have been detected in various types of cancers (Table 3) [25–27, 33, 35, 39, 40, 51–68]. The 4,977 bp deletion is one of the common mtDNA mutations detected in aging human tissues [69]. This deletion has 13-bp direct repeats flanking the 5’- and 3’-end breakpoints at np 8470/8482 and np 13447/13459, respectively. We first reported that this 4,977 bp deletion was largely accumulated in sun-exposed skin tissues and also occurred in the squamous cell carcinomas and precancerous skin tissues [63]. The 4,977 bp deletion of mtDNA was later detected in oral cancers and paired non-malignant oral tissues of patients with betel quid chewing history [60]. Although the 4,977 bp deletion of mtDNA has been frequently detected in various types of cancers (Table 3), the incidence and amount of the 4,977 bp-deleted mtDNA are significantly lower in the malignant tissues as compared with the paired normal tissues of cancer patients. We have suggested that during cancer progression the mtDNA with a deletion is decreased (diluted) as a result of clonal expansion of cell lineages that contain less or no mtDNA deletion. A study of micro-dissected tumor tissues further confirmed the lower incidence of 4977 bp mtDNA deletion in most tumors [51]. On the other hand, an increase in large-scale deletions of mtDNA was found in radiation-associated thyroid tumors [66].

A 50-bp deletion flanked by a 9-bp direct repeat in the D-loop region of mtDNA was identified in the tumor tissues of four patients with gastric cancer [67] and in one patient with hepatocellular carcinoma (HCC) [39]. The mtDNA deletion appeared to be homoplasmic and was largely accumulated in tumors, but not detected in paired normal tissues. In addition, a 294-bp deletion in the ND1 gene of mtDNA was detected in a patient with renal cell carcinoma [68]. Approximately 50% of the mtDNA molecules in the primary renal cell carcinoma contained this deletion, but none of the metastatic sites and unaffected tissues had this deletion in the ND1 gene. Taken together, these observations indicate that mtDNA deletions of different sizes occur and distribute in a stochastic manner in cancer tissues and tend to become more homogeneous, as a result of clonal expansion, during the progression of tumors.

### 2.3. Insertions

We identified two small insertions (~260 bp and ~520 bp) in the D-loop region of mtDNA in the cancerous tissues of one patient with gastric cancer [35]. The two insertions were characterized as tandem duplication and tandem triplication and the replicated DNA sequence is approximately 260-bp in size and is flanked by two poly-C stretches at np 303–309 and np 568–573, respectively. Such tandem duplications in the D-loop of mtDNA have also been detected in patients with mitochondrial myopathy [70–72], in somatic tissues of elderly subjects [73, 74], and in a Caucasian population with a specific mtDNA haplogroup [47]. In a further survey using a more sensitive molecular biological technique, we detected tandem duplication or triplication of mtDNA in approximately 5% of the patients with different types of cancers [75]. Moreover, it was found that the occurrence of the tandem duplications or triplications was highly associated with the presence of length variation at np 568 of the poly-C stretch in the D-loop of mtDNA [75]. Because the tandem duplication or triplication was also detected in the non-cancerous tissues of cancer patients and in about 4% of peripheral blood cells of normal subjects, we contend that the occurrence of tandem duplication or triplication in the D-loop region of mtDNA is not specific to cancers [75]. It was suggested that this type of mtDNA mutation is associated with the instability in the number of Cs starting at np 568 in the D-loop of mtDNA [75].

### 2.4. Copy number changes

Alterations in the copy number of mtDNA in cancer have been frequently found in various human cancers (Table 4) [26, 27, 29, 35, 39, 76–81]. A decrease in the content of mtDNA in cancer tissues has been reported in most of renal carcinomas [78, 79, 82], HCCs [39, 40, 77, 83], gastric cancers [35], and breast cancers [26, 27, 76]. However, an increase in the copy number of mtDNA was found in the majority of renal oncocytomas [78, 82], salivary gland oncocytomas [82], head and neck cancers [84], papillary thyroid carcinomas [76], colorectal cancers [29, 80], endometrial cancers [85], ovarian cancer [86], and prostate cancers [80]. These findings suggest that a change in the content of mtDNA is related to the type of cancers.

It was demonstrated that the decrease in mtDNA copy number is associated with the point mutations located near the replication origins in the D-loop of mtDNA in HCCs [39] and breast cancers [27]. In addition, it was revealed that altered mRNA expression of the genes involved in mitochondrial biogenesis, such as peroxisome proliferator-activated receptor γ coactivator-1α (PGC-1α) and mitochondrial single strand DNA binding protein (mtSSB) may be related to or responsible for the decrease in mtDNA copy number in HCCs [40]. These results suggest that somatic mutations in the D-loop of mtDNA and impairment in mitochondrial biogenesis may contribute to the decrease of mtDNA copy number in human cancers [29].

On the other hand, the increase in mtDNA content in cancers could be related to an increase of oxidative stress. It was demonstrated that enhanced oxidative stress can induce increases in mitochondrial mass and the copy number of mtDNA [87]. Moreover, an increase in the content of mtDNA was found to associate with elevated oxidative stress in aging tissues and in late passages of diploid human skin fibroblasts [88, 89]. Therefore, an increase in oxidative stress could lead to the increase of mitochondrial mass and mtDNA content in human cancers. In addition, the increase of mtDNA copy number could be a result of the feedback response that compensates for mitochondria with impaired respiratory function or mtDNA mutation in human cells [89].

## 3. Clinical correlations of somatic mtDNA alterations in cancers

### 3.1. Point mutations

Matsuyama and coworkers [41] found that in 202 patients with non-small cell lung cancer examined, the average mutation rate in the D-loop of mtDNA of patients at stage IIIB or stage IV was significantly higher than that of patients at lower clinical stages. Moreover, the stage IIIB or stage IV cancer patients carrying point mutations in the D-loop of mtDNA exhibited poorer prognosis compared with those free of the mtDNA mutations [41].

Tamori *et al*. [89] observed a higher frequency of D-loop mutations of mtDNA in poorly differentiated HCCs. However, there was no relationship between the occurrence of D-loop mutations in mtDNA and the age of cancer patients or between mtDNA mutation and hepatitis virus infection [90].

A study of 365 colorectal cancer patients revealed that the presence of D-loop mutations is a factor of poor prognosis in patients with colorectal cancers [91]. In the subgroup of patients with stage III colon cancer receiving fluorouracil-based adjuvant chemotherapy, the three-year survival rate for patients with D-loop mutation of mtDNA was significantly lower than those without D-loop mutations [91]. It was suggested that D-loop mutations of mtDNA are related to the resistance to adjuvant chemotherapy in patients afflicted with stage III colon cancers.

Recently, we analyzed the association between the clinicopathological features and D-loop mutations of mtDNA in 60 patients with breast cancers and found that the occurrence of D-loop mutations of mtDNA is associated with old onset age (≥ 50 years) and with the tumors that lacked the expressions of estrogen receptor and progesterone receptor, respectively [26]. Moreover, we noticed that the breast cancer patients harboring D-loop mutations of mtDNA had significantly poorer disease-free survival compared with those without mtDNA mutations [26]. These observations suggest that somatic mutation in the D-loop of mtDNA can be considered as a new prognostic marker for some types of cancers, and that mtDNA mutations may play a role in cancer progression and in response to anticancer drug treatment.

In contrast, a study of 109 patients with head and neck cancers revealed that the presence of D-loop mutations of mtDNA was not associated with the prognosis or the response of patients to neoadjuvant chemotherapy [36]. Moreover, no significant association was found between somatic mtDNA mutations and clinicopathological characteristics in esophageal cancer [31, 92], gastric cancer [35], lung cancer [16], and ovarian cancer [44], respectively.

### 3.2. Copy number changes

Yin *et al*. [40] reported that the copy number of mtDNA was frequently reduced in HCCs. Moreover, reduction in the copy number of mtDNA was more frequently observed in female patients with HCCs as compared with male patients with HCCs. This finding suggests that the differential alterations in the mtDNA copy number of cancer tissues of male and female patients may contribute to the differences in clinical manifestation, progression, and mortality rate between male and female HCC patients [40]. Yamada *et al*. [77] reported that mtDNA copy number was reduced in HCCs compared with the corresponding non-cancerous liver tissues, and that low mtDNA content of HCCs was significantly correlated with large tumor size and liver cirrhosis. Moreover, patients with lower mtDNA content in HCCs tended to show poorer 5-year survival compared with the patients with higher mtDNA content in HCCs, which suggest that decrease in the mtDNA content may be associated with malignancy of HCCs [77].

In gastric cancers, we analyzed the association between the clinicopathological features and the mtDNA content and found that a decrease of mtDNA content is significantly associated with ulcerated and infiltrating type (Borrmann’s type III) and diffusely thick type (Borrmann’s type IV) of gastric carcinomas [35]. Most patients with types III and IV gastric cancers, respectively, were found to have poor prognosis and lower 5-year survival rate after gastric resection. These results suggest that the reduction in the content of mtDNA may contribute to the malignancy and progression of gastric cancers [35].

A decrease in mtDNA copy number was also found to associate with an older onset age (≥ 50 years old) and a higher histological grade of breast cancer [27]. In addition, patients with reduced mtDNA content had significantly poorer disease-free survival and overall survival rate [27]. These results suggest that reduction in the content of mtDNA may be involved in neoplastic transformation or progression of breast cancers. However, no similar association was found in other studies of breast cancer patients [26,76].

The mtDNA content in ovarian carcinomas was found to be significantly higher than that in normal ovaries [86]. However, it was shown that the mtDNA content in the pathologically high-grade (poorly differentiated) ovarian cancer was lower than that of the low-grade (well differentiated) ovarian cancer [86]. These findings suggest that a decrease in the mtDNA content is associated with the progression of ovarian cancer.

Recently, a study of 153 colorectal cancer patients revealed that mtDNA content in colorectal cancers was higher than that in the corresponding non-cancerous colon tissues [80]. However, the mtDNA content was decreased in colorectal cancers with higher TNM stages and poorer differentiation [80]. The decrease in mtDNA content was correlated with a lower expression level of mitochondrial transcription factor A (mtTFA) or β subunit of the mitochondrial ATP synthase (β-F_1_-ATPase). It was suggested that mitochondrial dysfunction is associated with poor prognosis of colorectal cancer [80].

In contrast, it was observed in head and neck cancers that mtDNA content was increased with histopathologic grade from normal, moderate, dysplasia, severe dysplasia to invasive tumors [84]. The increase in mtDNA content was thought to be a feedback mechanism that compensates for a decline in respiratory function. In addition, Jiang *et al*. [93] reported that the mtDNA content in saliva from patients with primary head and neck squamous cell carcinoma was significantly higher than that of controls, and that an increase in the mtDNA content was associated with advanced tumor stage [93].

Based on the clinicopathologic correlations with somatic mtDNA alterations of cancers, these mtDNA alterations can potentially be used as a molecular prognostic indicator of cancers. Their correlations with poorer prognosis suggest that somatic mtDNA alterations in cancers may contribute to tumor recurrence and drug resistance in the process of cancer progression.

## 4. Reduced content of mitochondrial respiratory enzyme complexes in cancers

Simonnet *et al*. [94] reported that the contents of mitochondrial respiratory enzyme Complexes II, III, IV, and V in renal cell carcinoma were significantly lower than those in normal kidney tissues. In addition, the decrease in mitochondrial respiratory enzyme complexes was correlated with the aggressiveness of renal cell carcinoma, suggesting that a decrease in the OXPHOS capacity favors faster growth or increased invasiveness of cancers [94]. It was also found that the contents of the subunits of mitochondrial respiratory enzyme Complexes II and III were decreased in HCCs [40]. A concomitant reduction in the copy number of mtDNA and the content of mitochondrial respiratory enzymes was observed in HCCs, which suggests that the biogenesis of mitochondria may be repressed in HCCs.

The protein level of β-F_1_-ATPase was reduced in carcinomas of human liver, kidney, colon, breast, stomach, squamous esophagus and lung, which suggests that alteration of the bioenergetic function of mitochondria is a hallmark of these types of cancers [83,95]. Moreover, a reduction in the protein level of β-F1-ATPase in colon cancers was significantly correlated with both the time of recurrence of the disease and the survival of the patients [83]. These observations have led us to suggest that the metastases and recurrence of cancers may be linked to the down-regulation of the mitochondrial OXPHOS system.

## 5. mtDNA mutations/mitochondrial dysfunction in cancer formation/progression

Because β-F_1_-ATPase expression is down-regulated frequently in many types of cancers [83,95], pathogenic mutations in ATP synthase subunit 6 gene (ATPase 6) of mtDNA has provided a model to evaluate the role of defective mitochondrial ATPase in tumorigenesis and progression of cancers [96]. Petros *et al*. [97] introduced a pathogenic T8993G mutation in the ATPase 6 gene of mtDNA into the PC3 prostate cancer cell line through cytoplasmic transfer. They found that the tumor generated by transplantation of the cybrid carrying T8993G mutation was much larger than that produced by the transplantation of wild-type (T8993T) cybrid into the nude mice. Moreover, the tumors bearing the T8993G mtDNA mutation generated significantly more ROS. This study further substantiated that mtDNA mutation increased tumor growth in the prostate cancer.

Using similar cybrid transfer technique, Shidara *et al*. [98] constructed cybrids containing HeLa nucleus and the T8993G or T9176C mutation in the ATPase 6 gene of mtDNA. They found that the mtDNA mutations conferred an advantage in the early stage of tumor growth in nude mice. By transplanting a mixture (1:1) of mutant and wild type cybrids into the nude mice, they observed that the proportion of mutant mtDNA in the tumor was increased progressively, and eventually the mutant mtDNA entirely replace the wild-type mtDNA [98]. These findings provide an explanation of the observation that most of somatic mtDNA mutations in many tumors tend to become homoplasmic during progression. In addition, apoptosis was found to occur less frequently in the mutant cybrids in cultures as compared with wild-type cybrids, which suggests that the pathogenic mtDNA mutations might promote the growth of tumors by preventing apoptosis [98]. The mutant mtDNA in cybrids also exhibited resistance to cisplatin-induced apoptosis [98]. These results suggest that pathogenic mtDNA mutations may contribute to the progression of cancers and tolerance against anticancer drugs.

Enhanced resistance to anticancer drugs was also observed in cultured cancer cells upon depletion of mtDNA and in cancer cells with mitochondrial respiratory chain defects [99–104]. Park *et al*. [100] reported that mtDNA-depleted human SK-Hep1 hepatoma cells are resistant to ROS generated by menadione, paraquat, and doxorubicin treatments, respectively. They suggested that an adaptive increase in the expression of manganese superoxide dismutase (MnSOD) and other antioxidant enzymes renders cancer cells harboring mtDNA mutation to counteract oxidative stress or chemotherapeutic agents [100]. Li *et al*. [102] found that pretreatment of human hepatoma HepG2 cells and non-small cell lung cancer H1299 cells with chloramphenicol, an inhibitor of mitochondrial translation, rendered the cells resistant to mitomycin-induced apoptosis. The chloramphenicol- induced mitochondrial stress was shown to increase the expression of p21 and cause resistance to apoptosis through the p21-dependent pathway [102]. Shin *et al*. [103] investigated human colon cancer cells with 5-fluorouracil (5-FU) resistance and found that the 5-FU-resistant cells exhibited lower expression of the α subunit of ATP synthase and lower ATP synthase activity in the mitochondria of cancers. These findings suggest that down-regulation of ATP synthase in colorectal carcinomas may lead to 5-FU resistance. Moreover, Pelicano *et al*. [104] observed that mitochondrial respiration defects induced by mtDNA deletion or chemical inhibitors led to the activation of the Akt survival pathway through a NADH-mediated PTEN inactivation. The activation of the Akt pathway in cancer cells may contribute to the observed resistance to drugs.

MtDNA has also been shown to determine the hormone dependence in prostate or breast cancer cell lines [105,106]. Higuchi *et al*. [105] reported that an androgen- independent cell line, established by inoculation of the androgen-dependent LNCaP cell line into castrated mice, had greatly reduced content of mtDNA and an accumulation of large-scale deletions of mtDNA. The depletion of mtDNA from androgen-dependent LNCaP cells resulted in a loss of androgen dependence [105]. These results suggest that mtDNA defects play an important role in the development of androgen independence, which may contribute to the progression of prostate cancer. Moreover, Naito and colleagues [106] established hydroxytamoxifen-resistant breast cancer cells by growing human breast cancer cells MCF-7 in the presence of hydroxytamoxifen. They found that the mtDNA content was significantly reduced in the hydroxytamoxifen-resistant breast cancer cells. The mtDNA-depleted MCF-7 cells were established by long-term treatment with ethidium bromide and were also shown to be resistant to hydroxytamoxifen and ICI182780, respectively [106]. They further demonstrated that depletion of mtDNA induced by hormone therapy or other independent insults could trigger a shift to acquired resistance to hormone therapy in breast cancers.

In addition, the development of multidrug resistance (MDR) phenotype could be enhanced by mtDNA depletion [107,108]. Lee *et al*. [107] demonstrated that human HCT-8 colon cancer cells with decreased mtDNA content exhibited higher tolerance to doxorubicin or vincristine, and that mtDNA depletion induced an increase in the expression level of mRNA of multidrug resistance 1 (MDR1) gene and its translated P-glycoprotein [107]. These results suggest that mtDNA depletion may induce drug resistance through up-regulation of MDR1 gene expression in cancer cells. Ferraresi *et al*. [108] reported that when compared to parental cells, mtDNA-depleted human osteosarcoma 143B cells are less sensitive to apoptotic drugs including staurosporine, doxorubicine, daunomycin, and quercetin, respectively. Moreover, it was suggested that an increase in the content of reduced form of glutathione and overexpression of P-glycoprotein in the mtDNA-depleted cells may contribute to drug resistance [108].

In contrast, the mtDNA content in head and neck cancers appeared to increase with histopathologic grade from normal, moderate, dysplasia, severe dysplasia to invasive tumors [84]. Mizumachi *et al*. [109] created docetaxel-resistant cells from human laryngeal cancer HEp2 cells and found that the docetaxel-resistant cells had higher mtDNA contents. They also showed that an increase in the mtDNA content could induce acquired docetaxel resistance in head and neck cancer cells, and that mtDNA plays an important role in developing docetaxel resistance through the reduction of ROS generation by regulating the function of Fo-ATPase [109].

Human mtDNA mutations or mitochondrial dysfunction may contribute to tumor progression by enhancing the metastatic potential of cancer cells. Amuthan *et al*. [110] showed that partial depletion of mtDNA or treatment with mitochondrial specific inhibitors induced invasive phenotypes in non-invasive C2C12 myoblasts and human lung cancer A549 cells. It was also reported that OXPHOS dysfunction could modulate the invasive phenotype by transcriptional regulation of extracellular matrix-remodeling genes [111]. Using hybrid technology to replace the endogenous mtDNA in tumor cells, Ishikawa *et al*. [112] demonstrated that ROS-generating mtDNA mutation can enhance metastatic potential of tumor cells. It was also reported that mtDNA-depleted LNCaP and MCF-7 cells exhibited invasive phenotype by epithelial-mesenchymal transition during depletion of mtDNA [113].

Results from the above-mentioned studies support that mtDNA mutations or mitochondrial dysfunction in cancer cells may play an important role in tumorigenesis, development of drug resistance, and metastasis of cancers. However, it should be noted that in creating cybrids with an mtDNA mutation or in the depletion of mtDNA of some cancer cell lines, diminished tumorigenic phenotypes were observed. These include slow cell proliferation, loss of anchorage-independent growth and increased sensitivity to cytotoxic chemotherapy [114–117]. Mitochondrial dysfunction induced by mtDNA mutation (e.g., A8344G) or inhibitors of respiratory chain was shown to impair proliferation of some cancer cells [118]. The molecular mechanisms by which mtDNA alterations or mitochondrial OXPHOS impairment affect tumor formation and cancer progression warrant further investigation.

## 6. Potential Roles of Mitochondria-to-Nucleus Signaling in Human Cancers

The expression of nuclear genes can be modulated in response to changes in the mitochondrial respiratory function in human cells. Mitochondrial dysfunction may induce the activation of nuclear transcription factors in a response called “retrograde response” [119,120]. The mitochondria-to- nucleus retrograde signaling pathways have been described in yeast and mammalian cells [119,120]. In yeast, numerous studies have shown that a retrograde signaling pathway can act as a homeostatic or stress response mechanism to adjust metabolic activities according to the state of mitochondrial function [120]. The retrograde signaling in mammalian cells, also known as mitochondrial stress signaling, has been demonstrated in C2C12 skeletal myoblasts and in human lung carcinoma A549 cells [121,122]. Partial depletion of mtDNA or treatment with inhibitors of mitochondrial respiratory chain resulted in elevated cytosolic free Ca^2+^ and activation of different nuclear transcription factors, which in turn induced the expression of specific nuclear genes that are involved in the progression of cancers [110,122]. Moreover, the mitochondria-to-nucleus retrograde signaling could induce aggressive and invasive phenotypes and resistance to apoptosis [101,110].

On the other hand, mtDNA mutation-elicited respiratory chain deficiency could result in over-production of ROS. ROS-induced oxidative stress is involved in the expression and regulation of nuclear genes related to carcinogenesis [123]. Recent studies showed that a pathogenic point mutation in the ATPase 6 gene of mtDNA led to over-production of ROS and enhanced tumor growth [97, 98]. Moreover, it was demonstrated that ROS over-production caused by mtDNA mutation can increase the metastatic potential of tumor cells [112]. These observations support a mechanism by which ROS mediate mitochondria-to-nucleus retrograde signaling that contributes to cancer progression and metastasis.

A host of mtDNA mutations and mitochondrial dysfunction have been observed in various human cancers. It has been shown that mitochondrial stress signaling in different types of human cells can modulate the expression of nuclear genes involved in carcinogenesis and cancer progression. Communication between mitochondria and the nucleus may play an important role in the regulation of initiation and progression of cancers. Therefore, mitochondria and mtDNA may be good targets for development of drugs for treatment of cancers with certain metabolic alterations.

## 7. Metabolic Shift in Cancers

Although Warburg reported more than seven decades ago that an increase in the utilization of glucose for energy production is one of the distinct biochemical features of cancer cells, this phenomenon was not fully appreciated until 1990’s. In the past two decades there has been reemerging interest in the investigation of the mechanisms by which mitochondrial dysfunction and mtDNA mutations get involved in the pathophysiology and progression of cancers [124].

Pedersen’s group found that tumors that exhibited the most pronounced “Warburg effect” had functional mitochondria with the capacity to make ATP, but the mitochondrial content was reduced in cancer cells resulting in a decrease of net oxygen consumption capacity [125]. They further demonstrated that mitochondria-bound form of hexokinase (hexokinase 2, HK-2) plays a pivotal role in the “Warburg effect” [126]. In addition, the binding of HK-2 to voltage-dependent anion channel (VDAC) in the outer mitochondrial membrane facilitated the increase of glucose metabolism [127] and the inhibition of apoptosis [128,129] by allowing preferred access of HK-2 to ATP synthesized on the inner membrane of mitochondria.

The other lines of studies showed that the hypoxia microenvironment of tumor cells and the hypoxia-inducible factor (HIF) could contribute to the increase of gene expression and activities of some glycolytic enzymes [130,131]. Recently, it was further demonstrated that HIF-1-mediated expression of pyruvate dehydrogenase (PDH) kinase, which phosphorylates and inhibits the activity of PDH, is involved in the down-regulation of mitochondrial respiration [132,133]. These findings suggest that the hypoxic microenvironment of tumor cells and the activation of HIF-1 are involved in the enhanced utilization of glucose for energy production and reduced mitochondrial respiration.

It was reported that mutations in the genes encoding subunits of succinate dehydrogenase (SDH) and fumarate hydratase of the tricarboxylic acid (TCA) cycle were associated with pheochromocytoma, paraganglioma or renal cell carcinoma, and that SDH down-regulation was observed in gastric and colon carcinoma [134]. Recent evidence revealed that succinate, a TCA cycle metabolite, is accumulated due to SDH down-regulation and transmits an “oncogenic” signal from mitochondria to the cytosol [135]. Succinate may inhibit HIF-1α prolyl hydroxylase and lead to stabilization of HIF-1α under normoxic conditions. Thus, succinate can increase the expression of genes that facilitate glycolysis in cancer cells [134,135].

Several recent studies revealed that the products of some oncogenes (e.g., *ras*, *src*, *c-myc*, or *akt*) can up-regulate the gene expression and activities of some glycolytic enzymes [136–139]. The p53 protein was recently shown to regulate mitochondrial respiration [140] and glucose metabolism [141]. In addition, Dang *et al*. [142] have also systemically investigated the mechanism by which some oncogenes or tumor suppressor genes get involved in the modification of energy metabolism and confer a preference for glucose utilization on human cancers. They and other investigators showed that the tumors with greater metastatic potential tend to rely more on glycolysis for supply of energy. These findings are consistent with the observations that a wide spectrum of mtDNA alterations and mtDNA depletion occur in the pre-cancerous as well as cancerous tissues in patients with various types of cancers [26,29,35,39]. We believe that the decrease in mtDNA copy number and/or expression of genes involved in mitochondrial biogenesis will cause impairment in the mitochondrial OXPHOS system. It is easily rationalized that a decrease in mtDNA copy number may also lead to a low abundance and/or decreased activity of mitochondria in cancer tissues. Under such circumstances, the rapidly growing cancer cells will have to shift the energy supply from aerobic metabolism to glycolysis. This indeed happens in many cancers, especially in the highly metastatic cancers. We found a decrease in the mtDNA copy number and lower ROS-triggered damage to mtDNA in lung cancer tissues [143]. We suggest that the decrease in oxidative DNA damage might be the result of a decrease in the metabolic activity and less production of ROS in the mitochondria of cancer cells. The other reason is the increase of production of NADPH, the reducing equivalent for maintenance of the GSH pool, through the pentose phosphate pathway that is enhanced by increased utilization of glucose. The decrease in the copy number of mtDNA and oxidative DNA damage may indicate that cancer cells shift their energy supply from aerobic metabolism in mitochondria to glycolysis in the cytosol. The molecular mechanism underlying this phenomenon is under intensive studies.

## 8. Concluding Remarks

In the past decade, we and many other groups of investigators have demonstrated that mtDNA mutations occur in both cancerous and non-cancerous tissues of patients with various cancers. The sequence variations in the D-loop region, especially the D310 variations, are the most abundant point mutations of mtDNA in human cancers. The 4977 bp deletion is the most common large-scale deletion of mtDNA that occurs and accumulates in human cancers. Thus, mtDNA instability is a molecular hallmark of many types of human cancers. It was demonstrated that somatic D310 variations and alteration of the mtDNA copy number are correlated with the clinicopathological features or prognosis of some types of human cancers. Besides, it was found that a heterplasmy-to- homoplasmy shift of mtDNA generally occurs in the process of tumor progression. This may be caused by clonal expansion of cancer cells bearing certain mtDNA mutation(s) during the carcinogenesis and progression of cancers from the viewpoint of the replication and segregation of mtDNA. These mtDNA mutations as a whole may result in mitochondrial dysfunction due to decreased expression of mtDNA-encoded polypeptides and compromised function of respiratory enzyme complexes. Under such conditions, the affected cells will shift the reliance of ATP supply from aerobic metabolism to glycolysis, even in the presence of ample supply of oxygen. The cancer cells with defective mitochondria (and mutated mtDNA) also produce larger amounts of ROS and are thus exposed to higher oxidative stress. The mitochondria with higher oxidative stress may utilize the so-called retrograde signaling pathways to modulate the expression of nuclear genes involved in glycolysis and mitochondrial respiration and OXPHOS. This scenario, termed Warburg effect, may explain the observed increase in glucose utilization and higher lactate production in the formation and progression of cancers. Therefore, development of drugs that target to mitochondria or mtDNA may improve treatment of some types of human cancers in the future. In addition, it was recently shown that transformation of human mesenchymal stem cells is associated with an increase of OXPHOS [144]. Bioenergetic changes during transformation of stem cells might be quite different from those occur during the transformation of somatic cells. This may suggest that the mechanisms involved in the transformation of stem cells are different from that involved in differentiated cells transformation. The roles of the bioenergetic alterations of mitochondria in different types of cancers and cancer stem cells warrant further investigation.

## Figures and Tables

**Table 1.** Somatic mutations in mtDNA of human cancers.

Cancer type and case number	Somatic mutations in mtDNA of cancers			Reference
	Frequency (%)	% of mutations in the D-loop region	% of mutations in coding regions	
Bladder cancer				
N = 14	64	30	70	[10]
Brain tumor				
N = 15	40	61	39	[11]
Breast cancer				
N = 18	61	58	42	[12]
N = 19	74	81.5	18.5	[13]
N = 15	93	38	62	[14]
Colorectal cancer				
N = 10	70	0	100	[9]
Head & neck cancer				
N = 13	46	67	33	[10]
N = 83	49	25	75	[15]
Lung cancer				
N = 14	43	70	30	[10]
N = 55	60	32	68	[16]
Nasopharyngeal cancer				
N = 23	52	92	8	[17]
Oral cancer				
N = 18	78	77	23	[18]
Ovarian cancer				
N = 10	60	73	27	[19]
Pancreatic cancer				
N = 5	80	0	100	[20]
Renal cancer				
N = 8	62.5	17	83	[21]
N = 9	78	33	67	[22]
N = 15	47	29	71	[23]
Thyroid cancer				
N = 13	23	0	100	[24]
N = 66	68	51	49	[25]

**Table 2.** Somatic mutations in the D-loop region of mtDNA of human cancers.

Cancer type and case number	Somatic mutations in the D-loop of mtDNA		Reference
	Frequency (%)	% of mutations in D310	
Bladder cancer			
N = 14	36	17	[10]
Brain tumor			
N = 15	40	45.5	[11]
Breast cancer			
N = 18	39	71	[12]
N = 19	63	18	[13]
N = 15	53	29	[14]
N = 60	30	72	[26]
N = 59	42	45	[27]
Colorectal cancer			
N = 13	23	33	[28]
N = 25	40	90	[29]
Cutaneous neurofibromas			
N = 19	37	71	[30]
Esophageal cancer			
N = 37	5	50	[31]
N = 20	40	54	[32]
N = 21	33	13	[33]
Gastric cancer			
N = 8	37	25	[28]
N = 45	4	0	[34]
N = 31	48	67	[35]
Head & neck cancer			
N = 13	23	17	[10]
N = 109	21	76	[36]
N = 83	29	0	[15]
Hepatocellular carcinoma			
N = 19	68	61.5	[37]
N = 50	34	42	[38]
N = 61	39	41	[39]
N = 18	22	100	[40]
Lung cancer			
N = 14	36	14	[10]
N = 202	17	0	[41]
N = 31	23	57	[29]
N = 55	31	22	[16]
Oral cancer			
N = 30	57	22	[42]
N = 18	67	40	[18]
Ovarian cancer			
N = 10	50	18	[19]
N = 35	26	10	[43]
N = 44	57	45.5	[44]
Plexiform neurofibromas			
N = 18	50	18.5	[30]
Prostate cancer			
N = 16	87.5	16.7	[45]
N = 10	50	50	[46]
Renal cancer			
N = 8	12.5	0	[21]
N = 9	33	0	[22]
N = 15	27	0	[23]
Thyroid cancer			
N = 66	48.5	36	[25]

**Table 3.** MtDNA deletions in human cancers.

Deletions	Nucleotide position	Cancer types	Reference
4977 bp	8470–13447 or 8482–13459	Breast cancer	[26,51,52]
		Endometrial cancer	[53]
		Esophageal cancer	[33]
		Gastric cancer	[35,51,54]
		Head and neck cancer	[51,55]
		Hepatocellular carcinoma	[40,56–58]
		Lung cancer	[59]
		Oral cancer	[60,61]
		Renal cell carcinoma	[62]
		Skin cancer	[63–65]
		Thyroid cancer	[25,62,66]
50 bp	298–348 or 306–356	Gastric cancer	[67]
		Hepatocellular carcinoma	[39]
294 bp	3323–3588	Renal cell carcinoma	[68]

**Table 4.** Alterations in mtDNA content of human cancers.

Cancer type Case number	Alterations in mtDNA content		Reference
	Increase or Decrease	Frequency (%)	
Breast cancer			
N = 25	Decrease	80	[76]
N = 60	Decrease	63	[26]
N = 59	Decrease	78	[27]
Gastric cancer			
N = 31	Decrease	55	[35]
Hepatocellular carcinoma			
N = 38	Decrease	61	[39]
N = 31	Decrease	77	[77]
Renal cancer			
N = 13	Decrease	61	[78]
N = 37	Decrease	91	[79]
Colorectal cancer			
N = 25	Increase	40	[29]
N = 153	Increase	39	[80]
Lung cancer			
N = 31	Increase	48	[29]
Papillary thyroid cancer			
N = 20	Increase	65	[76]
Prostate cancer			
N = 9	Increase	78	[81]

## Abbreviations used:

ATPase6: ATP synthase subunit 6 gene in mtDNA;
β-F_1_-ATPase: the β-subunit of the mitochondrial ATP synthase;
HCC: hepatocellular carcinoma;
HIF: hypoxia-inducible factor;
HK-2: hexokinase 2;
MDR: multidrug resistance;
MnSOD: manganese superoxide dismutase;
mtDNA: mitochondrial DNA;
mtSSB: mitochondrial single strand DNA binding protein;
mtTFA: mitochondrial transcription factor A;
np: nucleotide position;
OXPHOS: oxidative phosphorylation;
PET: positron emission tomography;
PGC-1α: peroxisome proliferator-activated receptor γ coactivator-1α;
ROS: reactive oxygen species;
SDH: succinate dehydrogenase;
TCA: tricarboxylic acid.
